# Trends for invasive squamous cell neoplasia of the skin in Norway

**DOI:** 10.1038/sj.bjc.6690725

**Published:** 1999-10

**Authors:** T Iversen, S Tretli

**Affiliations:** The Cancer Registry of Norway, Montebello, Oslo, 0310, Norway; The Department of Obstetrics and Gynecology, University of Bergen, Norway

**Keywords:** skin, external ear, squamous cell carcinoma, incidence, anatomic sites, survival

## Abstract

Over the period 1966–1995, based on 11 662 patients, the incidence of squamous cell carcinoma of the skin increased three to four times in Norway mainly as a result of an increased number of localized tumours. In men, cancer of the auricle was the second most common site; in women the incidence was low. © 1999 Cancer Research Campaign


*British Journal of Cancer* (1999) **81**(3), 528–531
© 1999 Cancer Research Campaign
Article no. bjoc.1999.0725

Short Communication

### Trends for invasive squamous cell neoplasia of the skin in Norway

**T Iversen[1,2] and S Tretli[1]**

[1]The Cancer Registry of Norway, Montebello, 0310 Oslo, Norway; [2]The Department of Obstetrics and Gynecology, University of Bergen, Norway

**Summary** Over the period 1966–1995, based on 11 662 patients, the incidence of squamous cell carcinoma of the skin increased three to four times in Norway mainly as a result of an increased number of localized tumours. In men, cancer of the auricle was the second most common site; in women the incidence was low. © 1999 Cancer Research Campaign

**Keywords:** skin; external ear; squamous cell carcinoma; incidence; anatomic sites; survival

Most national studies of skin cancer have concerned malignant melanomas, with few on squamous cell carcinoma of the skin. In an earlier study from the Cancer Registry of Norway, basic data for the period up to 1988, for all the different types of skin cancer in Nordic countries, were presented (Magnus, 1991). Here, we present and discuss the population-based data for squamous cell carcinomas in Norway with special reference to trends in incidence, survival rate, and anatomic sites.

### MATERIALS AND METHODS

The study was restricted to patients with invasive squamous cell carcinoma of the skin that was histologically proven. In the above period we found only 14 men and 13 women with skin tumours diagnosed only by clinical examination, and these patients are not included in the study.

Incidence rates are presented per 100 000 per year, age-adjusted to the world standard population (Waterhouse et al, 1976). To evaluate the trends in incidence over the period 1966–1995, we used a simple log-linear model with changes presented in steps of 5-year periods.

The relative survival for a group is given by the ratio between observed and expected survival from all causes of death. The expected survival is estimated on the basis of survival data of the total female or male Norwegian population. In this way, relative survival can be interpreted as the proportion of patients alive at the end of the period if the cancer studied were the only cause of death.

A Poisson regression analysis was carried out to simultaneously examine the relationship between age at diagnosis, sex, period of diagnosis and localization.

### RESULTS

### Incidence

During the 30-year period 1966–95, 4888 women (median age 77 years, range 16–98) and 6774 men (median age 75 years, range 14–98) with squamous cell carcinoma of the skin were reported to the Cancer Registry of Norway. The annual age-adjusted incidence rates of invasive cancer per 100 000 women increased from 1.2 (all stages included) for the period 1966–1970 to 5.5 for the period 1991–1995 (Figure 1). Rates for men were 2.9 (all stages included) in the first period and 9.4 in the last. A total of 4647 women (median 78 years, range 16–98) and 6451 men (median 75 years, range 14–98) had localized lesions. Thus, during the period 1966–1995, the incidence of squamous cell carcinoma of the skin increased markedly in both women and men (4.6 and 3.2 times respectively). The increase is mainly caused, in both sexes, by an increased number of localized tumours. The incidence of primary metastatic disease was low and almost constant for the whole period (Figure 1).

### Anatomic localization

In both women and men, the highest proportion of invasive disease was found in the face and head region. In men, cancer of the auricle (external ear) was the second most common site of tumour

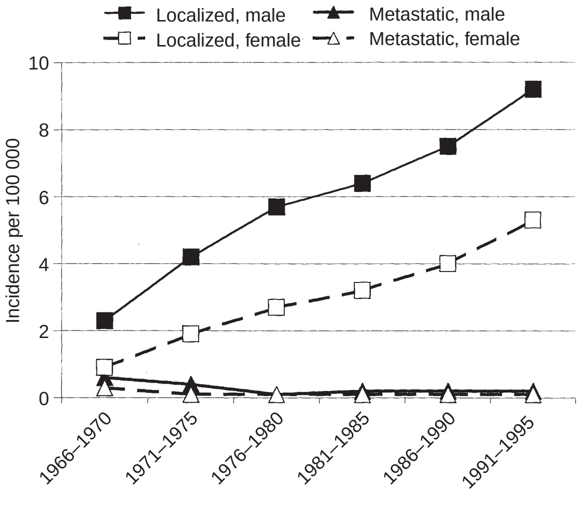

**Figure 1** Age-adjusted incidence rate with respect to the world standard population of squamous cell carcinoma of the skin in Norway 1966–1995

*Received 15 February 1999*
*Revised 29 March 1999*
*Accepted 31 March 1999*

*Correspondence to:* T Iversen, Hartmanns vei 28, 0284 Oslo, Norway

**Table 1** Incidence per 100 000 men or women per year of squamous cell carcinoma of the auricle by age and period of diagnosis

| Age | 1966–1970 | 1971–1975 | 1976–1980 | 1981–1985 | 1986–1990 | 1991–1995 |
|---|---|---|---|---|---|---|
| Males | | | | | | |
| 0–49 | 0.0 | 0.0) | 0.0 | 0.1 | 0.0 | 0.1 |
| 50–59 | 0.8 | 1.2 | 1.5 | 1.9 | 1.7 | 1.0 |
| 60–69 | 3.8 | 4.6 | 5.5 | 6.8 | 8.4 | 5.9 |
| 70–79 | 8.7 | 10.7 | 17.2 | 18.9 | 23.3 | 22.2 |
| 80+ | 28.3 | 36.1 | 42.4 | 41.3 | 53.5 | 53.7 |
| Crude rate | 1.4 | 1.9 | 2.6 | 3.1 | 3.8 | 3.5 |
| Total number of patients | 133 | 184 | 264 | 312 | 394 | 379 |
| Age-adjusted[1] | 0.9 | 1.1 | 1.5 | 1.7 | 2.0 | 1.7 |
| Number with localized tumour | 115 | 169 | 258 | 304 | 382 | 366 |
| Females | | | | | | |
| 0–49 | 0.1 | 0.1 | 0.0 | 0.0 | 0.0 | 0.1 |
| 50–59 | 0.3 | 0.1 | 0.3 | 0.4 | 0.3 | 0.2 |
| 60–69 | 0.6 | 0.3 | 0.2 | 0.5 | 0.5 | 0.5 |
| 70–79 | 1.4 | 0.1 | 0.8 | 0.5 | 1.1 | 0.5 |
| 80+ | 2.8 | 1.7 | 1.4 | 1.8 | 1.2 | 1.1 |
| Crude rate | 0.3 | 0.2 | 0.2 | 0.2 | 0.3 | 0.2 |
| Total number of patients | 30 | 15 | 17 | 23 | 27 | 22 |
| Age-adjusted[1] | 0.2 | 0.1 | 0.1 | 0.1 | 0.1 | 0.1 |
| Number with localized tumour | 21 | 11 | 16 | 16 | 20 | 18 |

[1]Age-adjusted incidence rate with respect to the world standard population.

(Figure 2). In the auricle, the incidence rate was low and constant in women (median age 71 years, range 27–94) (Table 1). Among men (median age 76 years, range 39–98), the age-adjusted incidence rate was 15–20 times higher, caused mostly by an increased number of patients with localized tumours.

The observed 5-yearly percentage increase was larger for women than for men, for all anatomic sites combined and most of the specific sites (Table 2). Only neck, shoulders, body and auricle deviated. However, the confidence intervals are broad, and the results should be interpreted with due care.

The Poisson regression analysis showed a significant increase in incidence by increasing age and period of diagnosis. There was a need for an interaction term between sex and anatomic localization to obtain an acceptable goodness of fit. Subanalyses for each anatomic localization showed a relationship between period of diagnosis and sex when age was taken into account similar with the results presented in Table 2.

The disease was localized with no sign of metastases among 93–98% of the women and men and this was not dependent on anatomic site of the tumour. (Twenty-two per cent of the metastases was confirmed by histology.) The only exception was cancer of the auricle with 95.7% of the tumours localized among men compared with 76.1% among women.

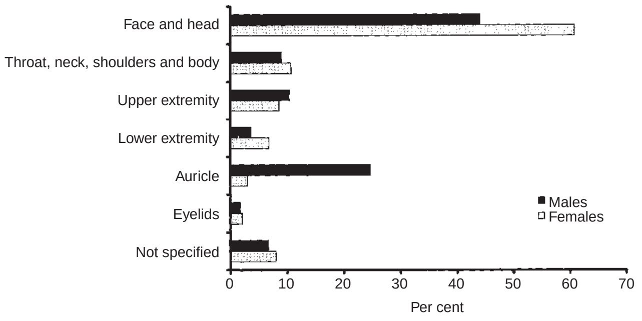

**Figure 2** Anatomic distribution of 4888 women and 6774 men with squamous cell carcinoma of the skin in Norway, 1966–1995

### Survival

The 5-year relative survival rate of all squamous cell carcinomas of the skin combined was high over the whole period and not influenced by age. In males, the 5-year relative survival rate (10-year in parentheses) increased from 86.1% (81.3%) in 1966–1970 (421 patients) to 91.4% (87.3%) in 1986–1990 (1494 patients). In females, the figures were 74.4% (67.7) in 1966–1970 (216 patients) and increased to 94.6% (88.6%) in 1986–1990 (1156 patients).

For men with carcinoma of the auricle, the 5-year relative survival rate was higher than 80% for the whole period. (The number of patients for each period is shown in Table 1.) However, among the women, the survival rate was low. In 1966–1970 the survival rate was 29.6% for all stages combined and 45.2% for those with localized disease. Even though the survival rate of women had improved, it was still inferior to that of the men. For the period 1986–1990, the 5-year survival rate in females was 84.8% and in men 97.8%. For the whole period 1966–1995, 32 women (24% of the females with carcinoma of the auricle; median age 61 years at the time of diagnosis, range 33–94) and 84 men (5% of the males with carcinoma of the auricle; median age 77 years at the time of diagnosis, range 43–95 years) died from cancer. For the other anatomic sites no such sex difference was observed in survival (data not shown). This difference in survival rate was independent of the region of residence in Norway.

*© 1999 Cancer Research Campaign*

**Table 2** Age-adjusted incidence rate per 100 000 per year (world standard population) of squamous cell carcinoma of the skin in Norway 1966–1995

| Localization | 1966–1970 | 1971–1975 | 1976–1980 | 1981–1985 | 1986–1990 | 1991–1995 | Median age in years (range) | Estimated 5-yearly increase (%) (95%CI) |
|---|---|---|---|---|---|---|---|---|
| Males | | | | | | | | |
| Total | 2.9 | 4.5 | 5.9 | 6.6 | 7.8 | 9.4 | 75 (14–98) | 24.5 (18.1–30.9) |
| Face, head | 1 | 1.8 | 2.3 | 2.9 | 3.3 | 4.4 | 76 (30–98) | 30.0 (21.9–38.1) |
| Neck, shoulders, body | 0.1 | 0.3 | 0.5 | 0.6 | 0.8 | 1.4 | 72 (27–98) | 60.2 (45.1–75.3) |
| Upper extremity | 0.4 | 0.5 | 0.6 | 0.8 | 0.8 | 0.9 | 74 (14–98) | 19.3 (14.9–23.7) |
| Lower extremity | 0.1 | 0.2 | 0.3 | 0.2 | 0.3 | 0.3 | 72 (22–97) | 22.0 (11.0–33.0) |
| Auricle | 0.9 | 1.1 | 1.5 | 1.7 | 2 | 1.7 | 76 (39–98) | 15.7 (7.9–23.5) |
| Eyelids | 0.1 | 0.1 | 0.1 | 0.1 | 0.2 | 0.1 | 72 (39–94) | 10.1 (–4.1–24.2) |
| Females | | | | | | | | |
| Total | 1.2 | 2.1 | 2.8 | 3.3 | 4.1 | 5.5 | 77 (16–98) | 32.5 (10.0–55.0) |
| Face, head | 0.5 | 1.2 | 1.7 | 2 | 2.3 | 3 | 76 (16–98) | 39.0 (22.5–55.5) |
| Neck, shoulders, body | 0.1 | 0.2 | 0.3 | 0.3 | 0.6 | 0.8 | 74 (20–97) | 49.2 (36.2–62.2) |
| Upper extremity | 0.1 | 0.2 | 0.2 | 0.4 | 0.5 | 0.6 | 76 (29–98) | 36.5 (28.7–44.3) |
| Lower extremity | 0.1 | 0.1 | 0.2 | 0.2 | 0.3 | 0.5 | 73 (29–98) | 31.5 (25.4–37.6) |
| Auricle | 0.2 | 0.1 | 0.1 | 0.1 | 0.1 | 0.1 | 71 (27–94) | –4.1 (–21.7–13.6) |
| Eyelids | 0 | 0 | 0.1 | 0.1 | 0.1 | 0.1 | 75 (25–96) | 40.0 (15.2–64.8) |

### DISCUSSION

The incidence of invasive squamous cell carcinoma of the skin has increased three and four times in men and women respectively in Norway during the 30-year period 1966–1995. Although the incidence differs among countries, several studies report a similar percentage increase of incidence to the one that we found in the present study (Green, 1992; Marks, 1995; Strom and Yamamura, 1997). Our data suggest that the age- and sex-specific rates are increasing. However, we have demonstrated that this increased incidence rate is restricted to localized tumours (Figure 1).

The major localization to the face, head, neck, shoulders and extremities, in our study, agrees with the results of others (Kaldor et al, 1993; Gray et al, 1997), and supports the theory of sunlight as an aetiologic factor (Gloster and Brodland, 1996; Strom and Yamamura, 1997). Changes in behaviour, such as clothing and sun-bathing, have increased exposure to the sun in Norway as well. The tumours on the auricle are 17 times more frequent in men compared with women and confirm another report (Kaldor et al, 1993). For the anatomic distribution, we found that 25% of the invasive cancers were localized to the external ear in men compared with 3% in women, which is similar to other results: 19% among men and 1% among women (Kaldor et al, 1993). We suggest that the reason for this sex difference is the longer hair of the women which protects the external ear from the sun.

The survival rate for squamous cell carcinoma is excellent except for cancer of the auricle, where the relative 5-year survival rate is lower (Østerlind et al, 1991; Rowe et al, 1992; Weinstock, 1994; Marks, 1995). However, the reduced survival rate is mainly restricted to women, those dying being younger at diagnosis compared with the men who died (median ages 61 and 77 years respectively). This reduced survival is mainly because more women than men had metastatic tumour at the time of diagnosis.

However, the survival rate in women with localized tumour was also lower than that in men with similar tumour localization, especially in the first part of our study. One reason might be that patient delay among women is longer than among men. Thus, female tumours might be more advanced at the time of diagnosis than those of the men. We suggest that the reason for this is the longer hair of the women. As the ear is hidden behind the hair, women may be more likely to 'wait and see', than men in whom the disease is visible to everyone.

### REFERENCES

Gloster HM and Brodland DG (1996) The epidemiology of skin cancer. *Dermatol Surg* **22**: 217–226

Gray DT, Suman VJ, Daniel Su WP, Clay RP, Scott Harmsen W and Roenigk RK (1997) Trends in the population-based incidence of squamous cell carcinoma of the skin first diagnosed between 1984 and 1992. *Arch Dermatol* **133**: 735–740

© 1999 Cancer Research Campaign

Green A (1992) Changing patterns in incidence of non-melanoma skin cancer. *Epithelial Cell Biol* **1**: 47–51

Kaldor J, Shugg D, Young B, Dwyer T and Wang YG (1993) Non-melanoma skin cancer: ten years of cancer-registry-based surveillance. *Int J Cancer* **53**: 886–891

Magnus K (1991) The Nordic profile of skin cancer incidence. A comparative epidemiological study of three main types of skin cancer. *Int J Cancer* **47**: 12–19

Marks R (1995) An overview of skin cancers. *Cancer* **75**: 607–612

Østerlind A, Hjalgrim H, Kulinsky B and Frentz G (1991) Skin cancer as a cause of death in Denmark. *Br J Dermatol* **125**: 580–582

Rowe DE, Carroll RJ and Day CL (1992) Prognostic factors for local recurrence, metastasis, and survival rates in squamous cell carcinoma of the skin, ear, and lip. *J Am Acad Dermatol* **26**: 976–990

Strom SS and Yamamura Y (1997) Epidemiology of nonmelanoma skin cancer. *Clin Plast Surg* **24**: 627–636

Waterhouse J, Muir C, Correa P and Powell J (1976) *Cancer Incidence in Five Continents*. International Agency for Research on Cancer: Lyon

Weinstock MA (1994) Epidemiologic investigation of nonmelanoma skin cancer mortality: the Rhode Island follow-back study. *J Invest Dermatol* **102**: 6S–9S

*© 1999 Cancer Research Campaign* 

